# The effect of gut microbiome and plasma metabolome on systemic sclerosis: a bidirectional two-sample Mendelian randomization study

**DOI:** 10.3389/fmicb.2024.1427195

**Published:** 2024-07-17

**Authors:** Shasha Xie, Qiming Meng, Lin Wang

**Affiliations:** ^1^Department of Rheumatology and Nephrology, The Affiliated Changsha Hospital of Xiangya School of Medicine, Central South University, Changsha, Hunan, China; ^2^Department of Rheumatology and Immunology, Xiangya Hospital, Central South University, Changsha, Hunan, China; ^3^Provincial Clinical Research Center for Rheumatic and Immunologic Diseases, Xiangya Hospital, Changsha, Hunan, China; ^4^National Clinical Research Center for Geriatric Disorders, Xiangya Hospital, Central South University, Changsha, Hunan, China

**Keywords:** systemic sclerosis, gut microbiota, plasma metabolites, Mendelian randomization, causally effect

## Abstract

**Background:**

Cellular and molecular biology, combined with research on the human microbiome and metabolome, have provided new insights into the pathogenesis of systemic sclerosis (SSc). However, most studies on gut microbiota (GM) and metabolome in SSc are observational studies. The impact of confounding factors and reverse causation leads to different insights. To shed light on this matter, we utilized Mendelian randomization (MR) to determine the causal effect of GM/metabolites on SSc.

**Methods:**

Based on summary-level data from genome-wide association studies, bidirectional Two-sample MR was conducted involving 196 GM, 1400 plasma metabolism, and 9,095 SSc. Inverse Variance Weighting (IVW) was mainly used for effect estimation.

**Results:**

Forward MR analysis found that three GM and two plasma metabolites are causally related to SSc. IVW results showed Victivallaceae (family) (OR, 1.469; 95%CI, 1.099–1.963; *p* = 0.009) and LachnospiraceaeUCG004 (genus) (OR, 1.548; 95%CI, 1.020–2.349; *p* = 0.04) were risk factor of SSc. Conversely, Prevotella7 (genus) (OR, 0.759; 95%CI, 0.578–0.997; *p* = 0.048)was a protective factor of SSc. The results on plasma metabolites indicated that Pregnenediol disulfate (C21H34O8S2) levels (OR, 1.164; 95%CI, 1.006–1.347; *p* = 0.041)was a risk factor of SSc, while Sphingomyelin (d18:1/19:0, d19:1/18:0) levels (OR, 0.821; 95%CI, 0.677–0.996; *p* = 0.045)was a protective factor of SSc. Reverse MR analysis did not find causally relationship between SSc and the above GM/plasma metabolites.

**Conclusion:**

Our results revealed the causally effect between GM/plasma metabolites and SSc. These findings provided new insights into the mechanism of SSc. In particular, we demonstrated Prevotella7 was a protective factor of SSc despite its controversial role in SSc in previous researches.

## Introduction

Systemic sclerosis (SSc) is an immune-mediated fibrotic disease characterized by autoimmunity, fibrosis of skin and internal organs, and vasculopathy (Fleischmajer et al., 1977; Prescott et al., 1992). The main clinical manifestations of SSc are skin and pulmonary fibrosis, gastrointestinal complications, renal crisis, Raynaud’s phenomenon, and pulmonary hypertension (Denton and Khanna, 2017; Volkmann et al., 2023). SSc has the highest mortality among all rheumatic diseases due to the lack of effective therapies (Steen and Medsger, 2007; Elhai et al., 2017). The mechanism of SSc remains unclear, with inflammation and immune dysregulation playing important roles (Fang et al., 2022). In recent years, cell and molecular biology combined with research on human microbiome, metabolome, genetics, and functional genomics have provided new insights into the pathogenesis of SSc.

The gastrointestinal tract is the second most commonly affected organ in SSc, following the skin. More than 90% of patients had gastrointestinal involvement during the course of disease, including constipation, malnutrition, diarrhoea, gastrointestinal bleeding, and faecal incontinence (Luquez-Mindiola et al., 2021). Gut microbes play a key role in human health. Changes in gut microbiota can cause immune and metabolic dysfunction, and influence the immune system and disease progression (Wu and Wu, 2012; Yoo et al., 2020). Several recent studies have reported changes in the gut microbiota of SSc. Volkmann et al. found that symbiotic bacteria (such as Faecalis, Clostridium, and Rikenella) were reduced in the colonic lavage fluid of 17 SSc patients, while pathogenic bacteria (including Clostridium, Prevotella, and γ-Proteus) increased (Volkmann et al., 2016). They found similar results in the faecal microbiome of SSc in two independent cohorts. In addition, among SSc patients with gastrointestinal symptoms, those with mild symptoms had an increased abundance of Clostridium compared to those with severe symptoms, and SSc patients with severe abdominal distension and diarrhoea had a higher abundance of Prevotella (Volkmann et al., 2017). However, two other observational studies (39SSc *VS* 17HC, Italy, 23SSc *VS* 19HC, Asia) found that Prevotella was reduced in the faecal microbiome of SSc patients (Natalello et al., 2017; Tan et al., 2023).

In terms of metabolomic research, some observational studies have also revealed the unique metabolic profile of SSc patients. Our team found that some serum metabolites (phloretin 2’-O-glucuronide, retinoyl b-glucuronide, all-trans-retinoic acid, and betaine) and metabolic pathways (starch and sucrose metabolism, proline metabolism, androgen and estrogen metabolism, and tryptophan metabolism) were dysregulated in new-onset SSc, but restored with treatment (Guo et al., 2023). A recent study reported enhanced kynurenine pathways in plasma and disorders of urea cycle and lipid metabolism in patients with SSc, such as down-regulation of tryptophan and up-regulation of kynurenine, dimethylarginine, and phenylacetylglutamine (Bögl et al., 2022).

We found that most studies of the gut microbiome and metabolome in SSc are observational studies. Observational studies have long been a cornerstone of epidemiological and medical research, providing valuable insights into the associations between various exposures and health outcomes. However, these studies come with several limitations that can compromise the validity and reliability of their findings. Some of these limitations include: Confounding Variables, Reverse Causation, Selection Bias, and Measurement Error (Hess, 2023). Mendelian randomization (MR) offers a promising solution to many of these limitations. MR is a method that uses genetic variants as instrumental variables to assess causal relationships between modifiable exposures and outcomes. This approach leverages the random assortment of genetic variants during meiosis, which mimics the properties of a randomized controlled trial (RCT), to overcome the limitations of observational studies. Some of the key advantages of MR include reduced confounding, mitigate reverse causation, mimicking randomization, and objective measurement (Zuber et al., 2022).

Hence, our study utilized MR to determine the causal relationship between gut microbes, metabolites, and SSc.

## Methods

### Study design

**Figure 1:**
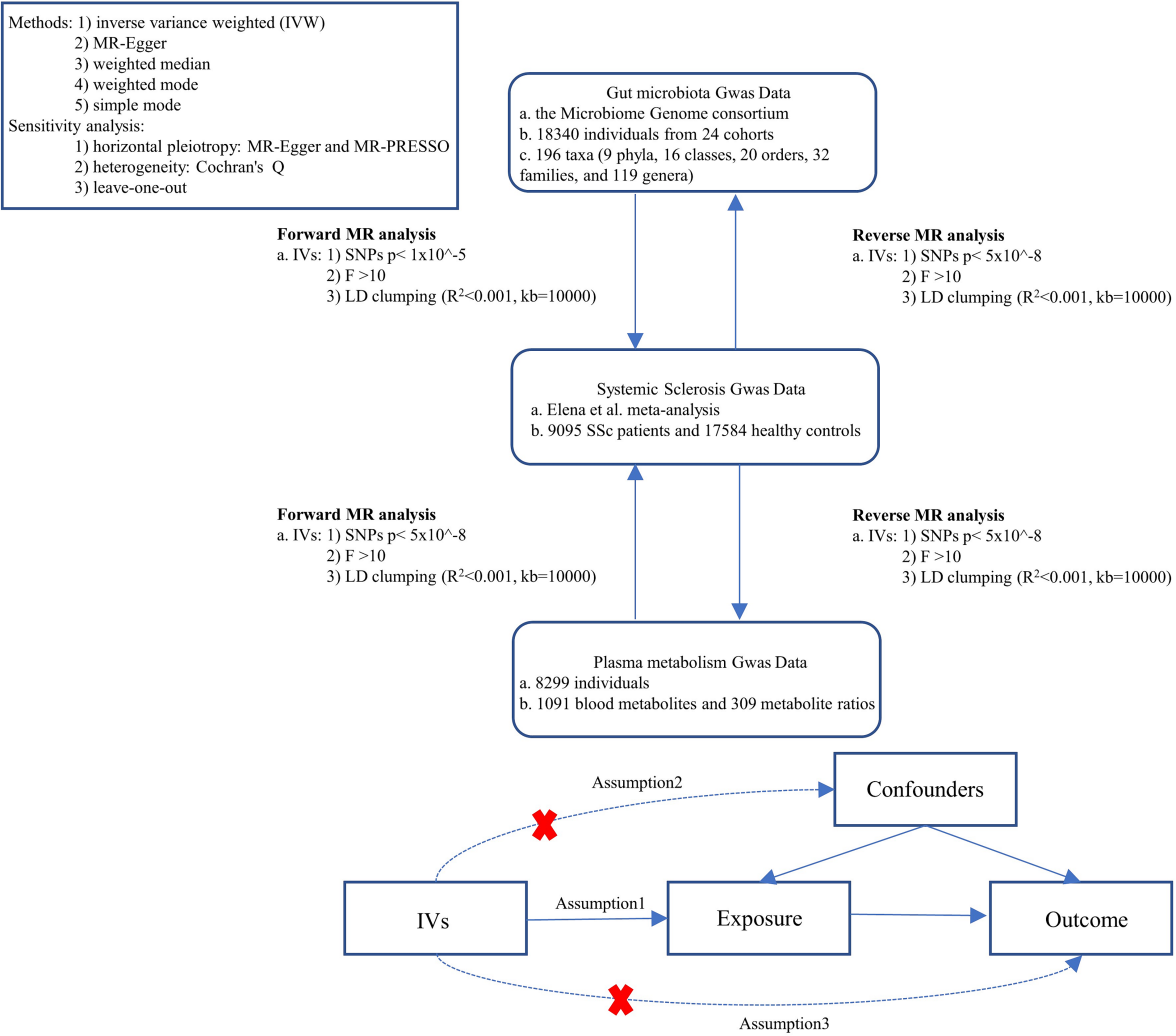
Study design. SSc, systemic sclerosis; IVs, instrument variables; GWAS, genome-wide association studies; IVW, inverse variance weighted; LD, linkage disequilibrium; MR, Mendelian randomization; SNPs, single nucleotide polymorphisms.

The study flowchart is illustrated in Figure 1. First, we obtained published GWAS summary data that included gut microbiota, plasma metabolism, and systemic sclerosis. Second, bidirectional Two-sample MR analysis were used to evaluate the causal relationship between gut microbes, plasma metabolites, and SSc. This study was following the STROBE-MR guidelines.

### Data source

Genetic associations for systemic sclerosis were obtained from genome-wide association study (GWAS) summary statistics in the meta-analysis by López-Isac et al. (2019). A total of 26,679 individuals (9,095 SSc patients and 17,584 healthy controls) were included in this meta-analysis, which combined 14 European-American SSc GWAS cohorts from 10 countries.

The GWAS summary data for human GM were acquired from the Microbiome Genome (MiBioGen) consortium (Kurilshikov et al., 2021). A total of 18,340 individuals from 24 cohorts were included in this study. 211 distinct microbial species, including 9 phyla, 16 classes, 20 orders, 35 families, and 131 genera were documented. 3 unidentified families and 12 unknown genera were excluded from the dataset, resulting a total of 196 GM for further analysis.

The GWAS data for plasma metabolites were retrieved from GWAS Catalog (GCST90199621-GCST90204603), accessible at: http://ftp.Ebi.ac.uk/pub/databases/gwas/summary_statistics/. The plasma metabolite data comprised 1,091 blood metabolites and 309 metabolite ratios.

### Selection of genetic instruments

To investigate potential causal links and associations between GM, plasma metabolites, and SSc, it is essential to select valid instrumental variables (IVs) that satisfy three key assumptions: (Prescott et al., 1992) the correlation hypothesis, (Fleischmajer et al., 1977) the exclusivity hypothesis, and (Volkmann et al., 2023) the independence assumption (Zuber et al., 2022). IVs were selected based on the following criteria: (Prescott et al., 1992) Statistically significant SNPs (SSc, *p* < 5 × 10^−8; GM, *p* < 1 × 10^−5; Plasma metabolites, *p* < 5 × 10^−8) are considered potential IVs; (Fleischmajer et al., 1977) To ensure the independence of each SNP, we applied a linkage disequilibrium (LD) factor (*R*^2^) of 0.001 and a clumping window width (kb) of 10,000, only the exposures with at least 3 IVs presenting *F* > 10 were left for MR (Arnold et al., 2015).

### Mendelian randomization analysis

MR analysis was conducted using five methods such as the inverse variance-weighted (IVW), MR-Egger, weighted median, weighted mode, and simple mode. MR-Egger regression and MR-PRESSO were used to assess the horizontal pleiotropy. In addition, Cochran’s Q test was utilized to quantify the heterogeneity of IVs. Further, to identify potentially heterogeneous SNPs, a “leave-one-out” analysis was performed by ignoring each tool for analyzing SNPs in turn (Verbanck et al., 2018).

All statistical analyses were performed using R version 4.2.1. MR analyses were performed using the TwosampleMR (version 0.5.7).

## Results

### Bidirectional causal association of gut microbiota and SSc

**Figure 2:**
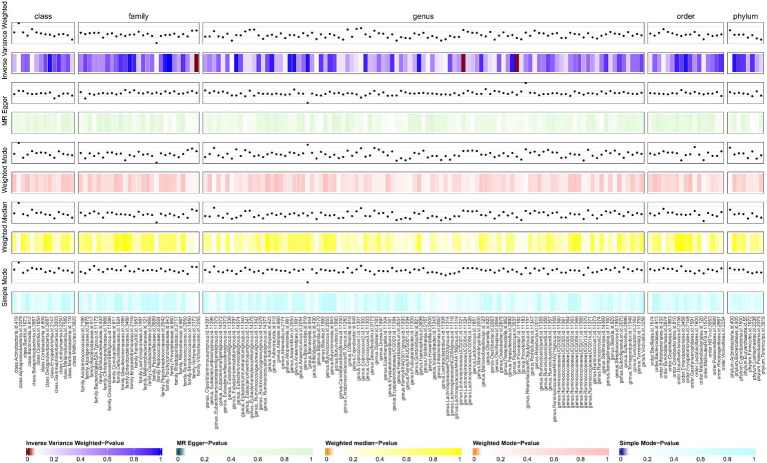
The result of gut microbiota (GM) on systemic sclerosis. This diagram shows the result of IVW, MR-Egger methods, weighted media, weighted mode, and simple mode from top to bottom. The classification of GM was based on class, family, genus, order, and phylum. The varying shades of color represent the magnitude of the *p* values. MR, Mendelian randomization; IVW, inverse variance-weighted. *p* < 0.05.

**Figure 3:**
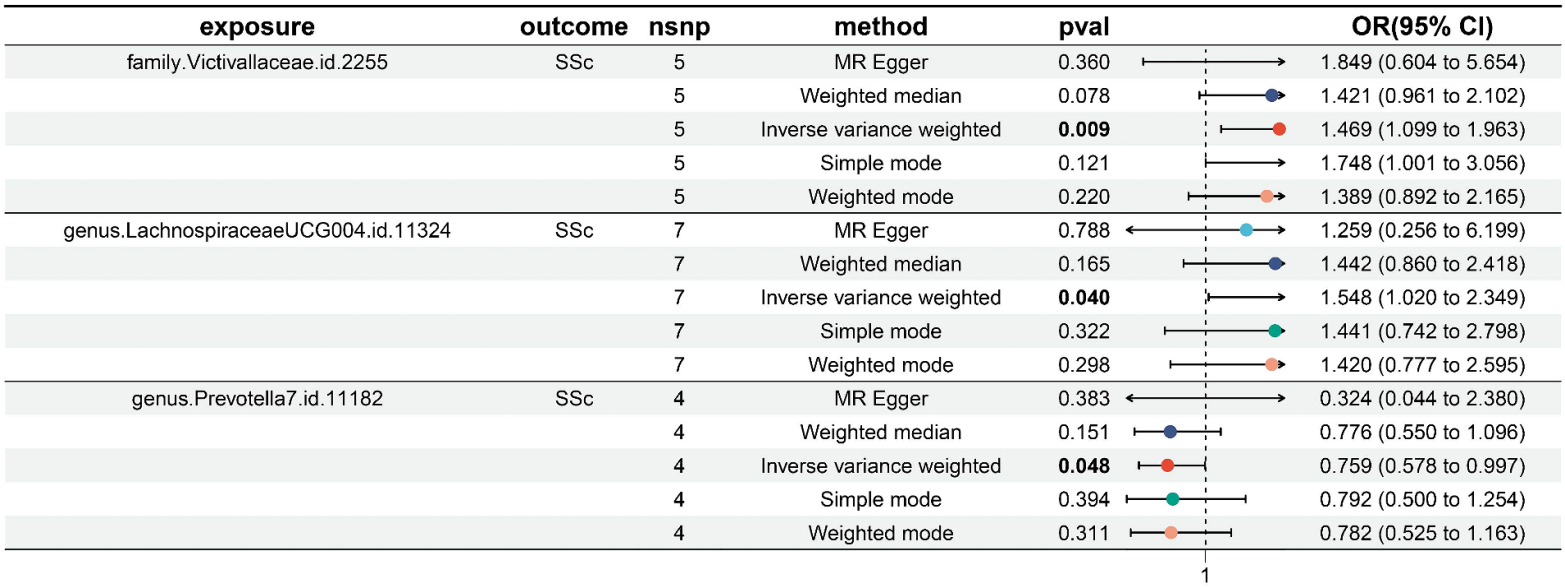
Forest plot to visualize the causal effects of GM to SSc. GM, gut microbiota; SSc, systemic sclerosis; OR, odds ratio; CI, confidence interval. *p* < 0.05.

We screened 877 SNPs as instrumental variables from 196 gut microbiota. The results of the MR analysis for IVs are shown in Supplementary Table S1. The results of IVW indicated Victivallaceae (family) (OR, 1.469; 95%CI, 1.099–1.963; *p* = 0.009) and LachnospiraceaeUCG004 (genus) (OR, 1.548; 95%CI, 1.020–2.349; *p* = 0.04) were associated with a higher risk of SSc. Conversely, Prevtella7 (genus) (OR, 0.759; 95%CI, 0.578–0.997; *p* = 0.048)was correlated with a reduced risk of SSc. The other four methods are consistent with the direction of the IVW beta value (Figures 2, 3; Supplementary Table S2).

Several IVs in the MR leave-one-out sensitivity analysis had effect values that span zero within the 95% CI, suggesting potential instability in the findings (Supplementary Figure S1). However, Cochran’s Q-test showed no significant heterogeneity for these IVs, the MR-Egger regression intercept analysis found no horizontal pleiotropy, and the MR-PRESSO test showed no outliers (Supplementary Table S3). Overly stringent filtering criteria may inadvertently exclude some valid positive findings. Therefore, we continue to confirm that the causal relationships of the above microbial taxa are valid.

Reverse MR analysis showed SSc was associated with the changes of 12 types of gut bacteria, but not the three above (Supplementary Figure S2; Supplementary Table S5).

### Bidirectional causal association of plasma metabolites and SSc

**Figure 4:**
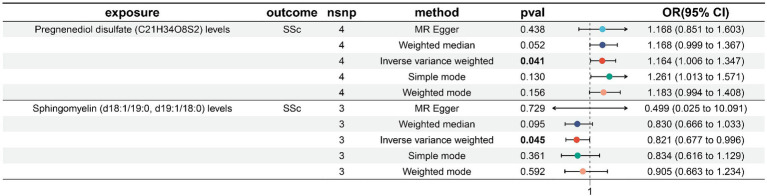
Forest plot to visualize the causal effects of plasma metabolites to SSc. SSc, systemic sclerosis; OR, odds ratio; CI, confidence interval. *p* < 0.05.

We screened 502 SNPs as instrumental variables from 1,400 plasma metabolites. The results of the MR analysis for IVs are shown in the Supplementary Table S7. The results of IVW indicated Pregnenediol disulfate (C21H34O8S2) levels (OR, 1.164; 95%CI, 1.006–1.347; *p* = 0.041) were associated with a higher risk of SSc. Conversely, Sphingomyelin (d18:1/19:0, d19:1/18:0) levels (OR, 0.821; 95%CI, 0.677–0.996; *p* = 0.045)was correlated with a reduced risk of SSc. The other four methods are consistent with the direction of the IVW beta value (Figure 4; Supplementary Table S8).

Several IVs in the MR leave-one-out sensitivity analysis had effect values that span zero within the 95% CI, suggesting potential instability in the findings (Supplementary Figure S3). However, *p* values indicating heterogeneity among the plasma metabolism mentioned above were > 0.05. MR-Egger regression intercept analysis found no horizontal pleiotropy, and the MR-PRESSO test showed no outliers (Supplementary Table S9). Overly stringent filtering criteria may inadvertently exclude some valid positive findings. Therefore, we continue to confirm that the causal relationships of the aforementioned plasma metabolism are valid.

Reverse MR analysis showed SSc was associated with the disturbances levels of 46 plasma metabolites, while no significant causal association was found with the two above (Supplementary Figure S4; Supplementary Table S11).

## Discussion

To our knowledge, this is the first bidirectional Two-sample MR Study exploring the causal relationship between gut microbes, plasma metabolites, and systemic sclerosis. Systemic sclerosis is an immune-mediated fibrotic disease in which almost all patients have gastrointestinal involvement and occur early in the course of the disease, suggesting that there may be a great connection between gut microbiota, immune abnormalities, and SSc (Denton and Khanna, 2017; Luquez-Mindiola et al., 2021; Kim et al., 2022). Our results showed that the family Victivallaceae and the genus LachnospiraceaeUCG004 significantly increased the risk of SSc, while Prevotella7 had a protective effect against SSc.

Lachnospiraceae play a central role in the gut microbiota, but their involvement in autoimmune or fibrotic diseases is controversial. The abundance of Lachnospiraceae is reduced in patients with Sjögren’s syndrome (SS) (Yang et al., 2022). The pathogenesis of SS was related to the reduction of butyrate, a metabolite of Lachnospiraceae, which reduces the differentiation of Treg cells and the secretion of the anti-inflammatory factor IL-10 (Kim et al., 2021). While increased Lachnospiraceae promotes the progression of rheumatoid arthritis, Lachnospiraceae A4 can promote Th1 and Th17 polarization and downregulate Th2 responses (Liu et al., 2016). Lachnospiraceae were more abundant in patients with non-alcoholic fatty liver disease or significant liver fibrosis but decreased in patients with primary biliary sclerosing cholangitis (PSC) (Shen et al., 2017). In PSC animal models, colonization of Lachnospiraceae mediates its anti-fibrotic effects through the production of short-chain fatty acids (Awoniyi et al., 2023). In SSc, studies have reported an increased abundance of unclassified Lachnospiraceae, which to some extent supports our findings (Patrone et al., 2017).

Prevotella is associated with a healthy plant-based diet and can function as a “probiotic” in the human body (Yeoh et al., 2022). The abundance of the Prevotella genus decreases in the gut microbiome of patients with multiple sclerosis (MS), and the supplementation of Prevotella effectively suppressed immune disease in preclinical MS models (Shahi et al., 2019). However, in rheumatoid arthritis, studies reported that the expression of Prevotella increases and promotes inflammatory responses by mediating Th17 and Th1 cell immunity. *Prevotella copri* was associated with disease severity in patients with new-onset RA (Scher et al., 2013; Jiang et al., 2022). The reported abundance of Prevotella in different SSc cohorts varies due to differences in sample size, region, diet and methods of gut microbiota acquisition, leading to different insights into the role of Prevotella in SSc. Volkmann et al. found that Prevotella was more abundant in SSc patients and is associated with more severe lower gastrointestinal symptoms, suggesting that it may be involved in the pathogenesis of SSc as a pathogenic bacterium (Volkmann et al., 2016, 2017). However, the two other cohorts found that Prevotella expression was reduced in patients with SSc, and in another cohort of SSc patients with gastrointestinal involvement who received probiotic treatment, increased Prevotella abundance was associated with improvement in gastrointestinal symptoms (Natalello et al., 2017; Patrone et al., 2017; Low et al., 2019). Our analysis included 14 SSc cohorts from 10 European-American countries and found that the genus Prevotella7 is a protective factor for SSc. Organ fibrosis is the hallmark of SSc. Respiratory failure caused by pulmonary fibrosis is the main cause of death in SSc patients (Tyndall et al., 2010; Denton and Khanna, 2017). TGF-β/Smad signaling pathway and cytokine IL-6 are crucial in SSc fibrosis (Varga and Pasche, 2009; Denton et al., 2018). Tocilizumab, a drug that targets IL-6R, has shown beneficial improvements in skin and pulmonary fibrosis in SSc (Denton et al., 2018). Previous studies shown that in other fibrotic diseases, Prevotella can inhibit the TGF-β/Smad signaling pathway and reduce the release of IL-6 to improve fibrosis (Bertelsen et al., 2021; Shen et al., 2023). Therefore, it is important to investigate the protective mechanism of Prevotella in SSc for the prevention and treatment of SSc.

Altered metabolic pathways in SSc patients are associated with gut dysbiosis, inflammation, vasculopathy, and fibrosis. We found that Pregnenediol disulfate (C21H34O8S2) levels were associated with a higher risk of SSc, while Sphingomyelin (d18:1/19:0, d19:1/18:0) levels were protective against SSc. Sphingomyelin’s metabolite sphingosine-1-phosphate (S1P) can reduce vascular leakage and intra-alveolar coagulation and inhibit pulmonary fibrosis through the S1P-S1P1 signaling pathway (Shea et al., 2010). However, in skin tissue, S1P can promote SSc dermal fibrosis through the TGF-β/Smad pathway (Xin et al., 2004; Radeke et al., 2005). Another metabolite, dihydrosphingosine 1-phosphate (dhS1P), has the opposite effect of S1P in skin tissue, inhibiting the TGF-β/Smad pathway in a PTEN-dependent manner, thereby reducing collagen production (Bu et al., 2008). Moreover, in a study on plasma phospholipid metabolism and clinical manifestations of SSc, it was found that the expression of Sphingomyelin was significantly decreased in patients with severe clinical symptoms (diffuse skin lesions, higher skin thickness scores, and duodenal ulcers) (Geroldinger-Simić et al., 2021).

Though our research obtained significant results, several limitations should be noted. First, Our study was based on European and American cohorts, and the results should be applied cautiously when it comes to other populations. Second, the results of MR cannot explain the biological mechanism behind the causal relationship, and further basic experiments are needed to demonstrate our findings.

## Data availability statement

The original contributions presented in the study are included in the article/Supplementary material, further inquiries can be directed to the corresponding authors.

## Author contributions

SX: Resources, Software, Supervision, Writing – original draft. QM: Formal analysis, Investigation, Resources, Software, Supervision, Writing – review & editing. LW: Formal analysis, Methodology, Software, Writing – review & editing.
