# Supplementary material for: The effect of gut microbiome and plasma metabolome on systemic sclerosis: a bidirectional two-sample Mendelian randomization study
Source: Front Microbiol. 2024 Jul 17;15:1427195. doi: 10.3389/fmicb.2024.1427195 (PMC11288946; doi:10.3389/fmicb.2024.1427195)
Supplement: Supplementary file 1 [file Table_1.DOCX]

Supplement Figures


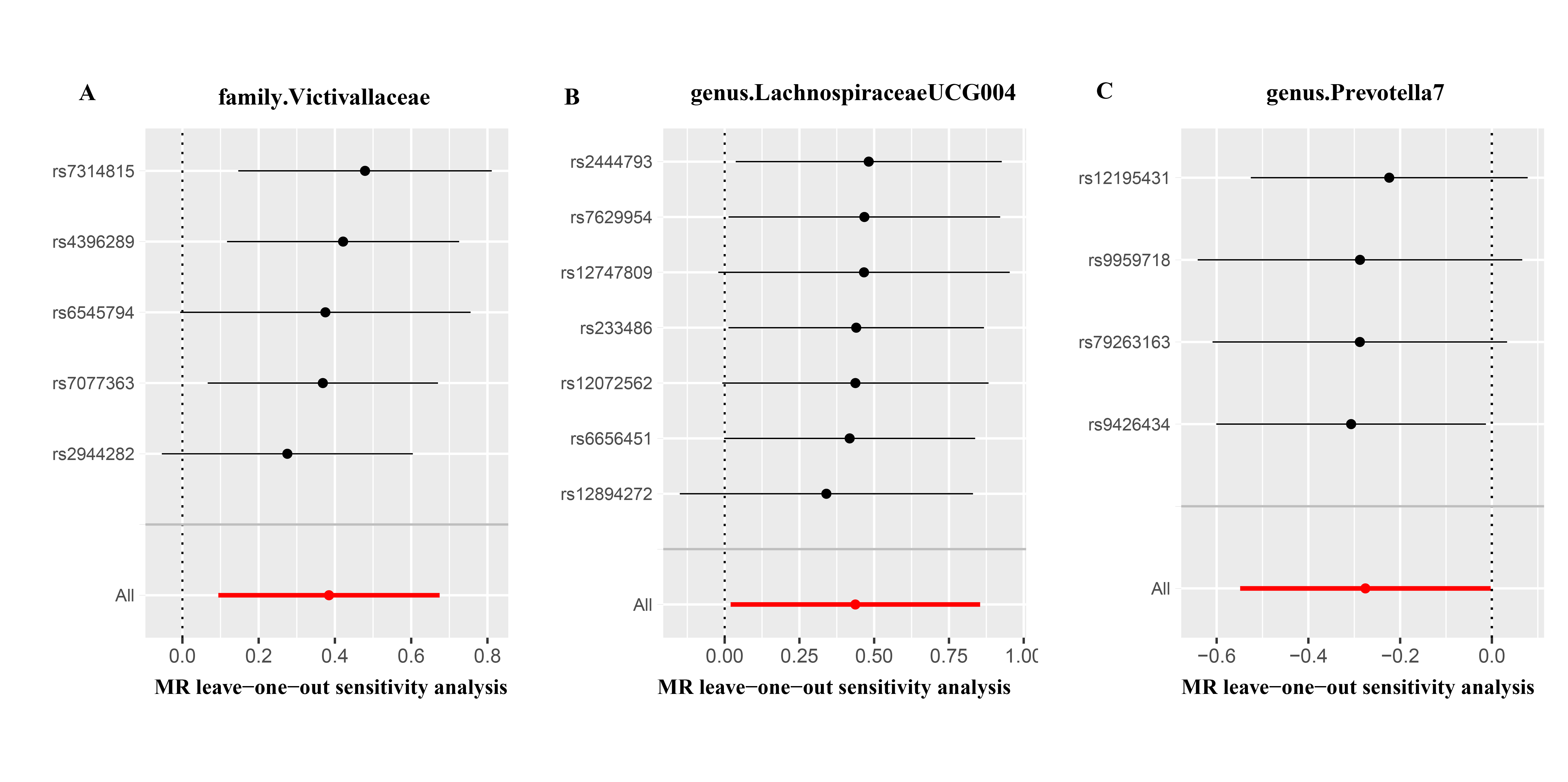


**Figure S1**. MR leave-one-out sensitivity analysis for gut microbiota abundance on SSc. (A) Leave-one-out sensitivity analysis of the effect of Victivallaceae on SSc; (B) Leave-one-out sensitivity analysis of the effect of LachnospiraceaeUCG004 on SSc; (C) Leave-one-out sensitivity analysis of the effect of Prevtella7 on SSc;





**Figure S2**. Forest plot to visualize the causal effects of SSc to GM. SSc, Systemic sclerosis; GM, gut microbiota; OR, odds ratio; CI, confidence interval. *P* < 0.05.


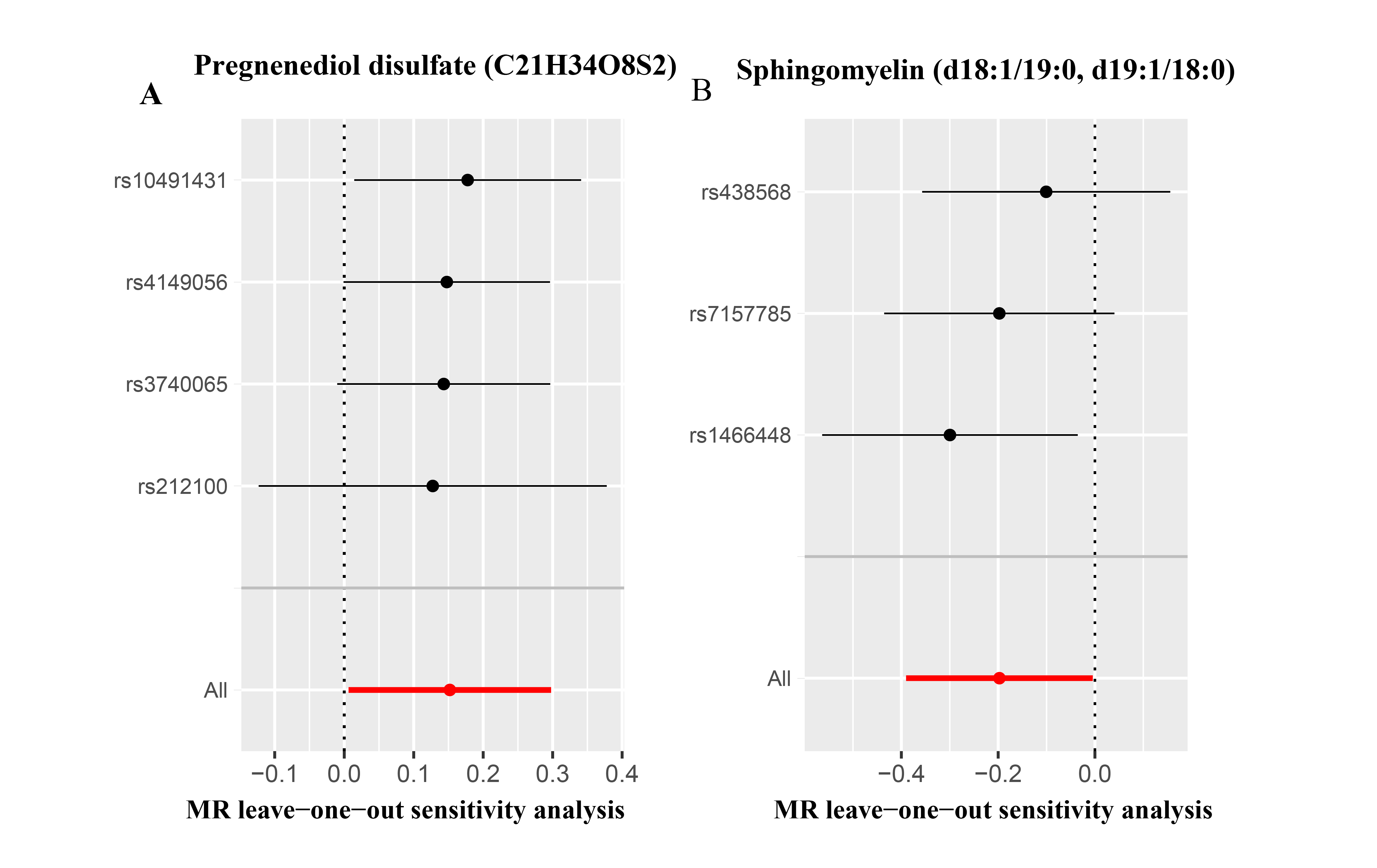


**Figure S3**. MR leave-one-out sensitivity analysis for plasma metabolites on SSc. (A) Leave-one-out sensitivity analysis of the effect of Pregnenediol disulfate (C21H34O8S2) on SSc; (B) Leave-one-out sensitivity analysis of the effect of Sphingomyelin (d18:1/19:0, d19:1/18:0) on SSc;


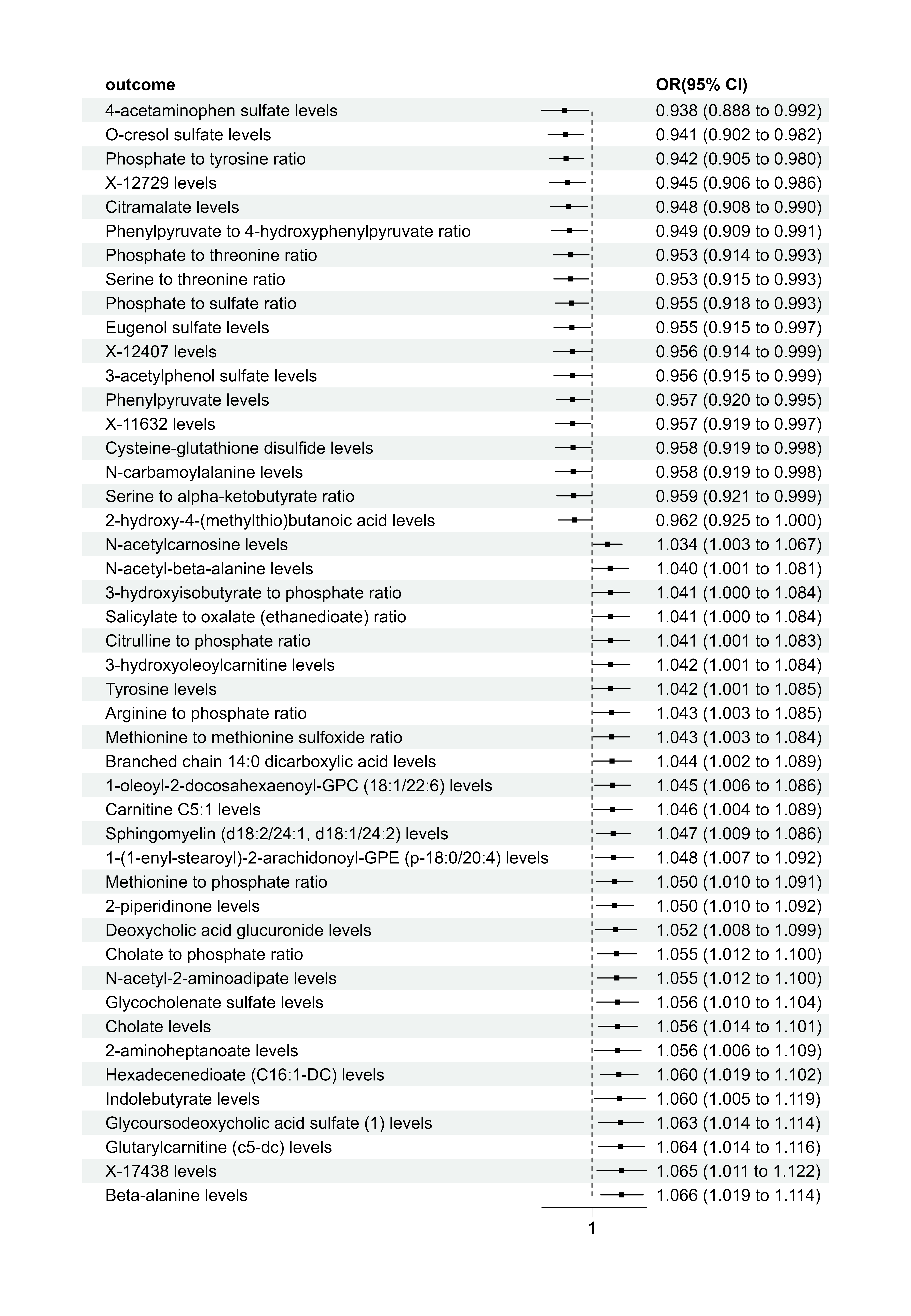


**Figure S4**. Forest plot to visualize the causal effects of SSc to plasma metabolites. The horizontal bars correspond to the estimated OR with 95% CI using the IVW method for SSc on plasma metabolites. SSc, Systemic sclerosis; OR, odds ratio; CI, confidence interval; IVW, Inverse Variance Weighting. *P* < 0.05.
